# Architecture of the NADPH oxidase family of enzymes

**DOI:** 10.1016/j.redox.2022.102298

**Published:** 2022-03-18

**Authors:** Blessing C. Ogboo, Uriy V. Grabovyy, Aniket Maini, Scott Scouten, Albert van der Vliet, Andrea Mattevi, David E. Heppner

**Affiliations:** a Department of Chemistry, University at Buffalo, The State University of New York, Buffalo, NY, USA; b Department of Pathology and Laboratory Medicine, Robert Larner, M.D. College of Medicine, University of Vermont, Burlington, VT, USA; c Department of Genetics and Microbiology, University of Pavia, Italy; d Department of Pharmacology and Therapeutics, Roswell Park Comprehensive Cancer Center, Buffalo, NY, USA

## Abstract

The NADPH Oxidases (NOX) catalyze the deliberate production of reactive oxygen species (ROS) and are established regulators of redox-dependent processes across diverse biological settings. Proper management of their activity is controlled through a conserved electron transfer (ET) cascade from cytosolic NADPH substrate through the plasma membrane to extracellular O_2_. After decades-long investigations of their biological functions, including potential as drug targets, only very recently has atomic-resolution information of NOX enzymes been made available. In this graphical review, we summarize the present structural biology understanding of the NOX enzymes afforded by X-ray crystallography and cryo-electron microscopy. Combined molecular-level insights predominantly informed by DUOX1 full-length Cryo-EM structures suggest a general structural basis for the control of their catalytic activity by intracellular domain-domain stabilization.

## 1 Introduction

The NADPH Oxidase (NOX) family of enzymes catalyze the reduction of O_2_ to reactive oxygen species, superoxide or hydrogen peroxide, coupled to the oxidation of NADPH [[1], [2], [3]]. The seven mammalian enzyme isoforms in the NOX Family (NOX1-5, DUOX1-2) are often found uniquely expressed in specific cell types and tissues highlighting their particular roles in specific functional circumstances [4,5], and relevance in disease [[6], [7], [8], [9], [10], [11]]. While much is known about their biological functions, considerably less is understood concerning the structural basis by which their activity is controlled. With the first reports of atomic-resolution structures of NOX5 [12] and DUOX1 enzymes [13,14], molecular-level information has very recently informed the structural basis for their regulation (Table 1). This structure-focused graphical review presents the current understanding of protein domain architecture and the molecular nature that governs NOX enzyme activity.

**Table 1:** Summary of deposited structures of NOX Family enzymes.

NOX Enzyme	Organism	PDB	Overall High resolution (Å)	Domains	Method	Notes	Citation
NOX2	*Homo sapiens*	3A1F	2.00	DH (NBL only)	X-ray crystallography	–	–
NOX5	*Cylindrospermum stagnale*	5O0X	2.20	DH	X-ray crystallography	C-terminal Mutant binds to the NADPH binding side	[12]
NOX5	*Cylindrospermum stagnale*	5O0T	2.05	TM	X-ray crystallography	–	[12]
NOX5-Calmodulin		**6SZ5**	2.23	–	X-ray crystallography		[16]
DUOX1-DUOXA1	*Mus musculus*	6WXU	2.70	TM, PHD	Cryo-EM	dimer-of-dimer inactive form	[13]
DUOX1	*Mus musculus*	6WXR	3.20	TM PHD DH	Cryo-EM	dimer no NADPH	[13]
DUOX1	*Mus musculus*	6WXV	3.30	TM PHD DH (NADPH diff)	Cryo-EM	dimer with bound NADPH active form	[13]
DUOX1	*Homo sapiens*	7D3E	2.80	TM PHD DH PHLD EF	Cryo-EM	Dimer-of-dimer low-calcium inactive form	[14]
DUOX1	*Homo sapiens*	7D3F	2.60	TM PHD DH PHLD EF	Cryo-EM	Dimer-of-dimer high-calcium active form	[14]

## 2 Overall primary structure

**Fig. 1:**
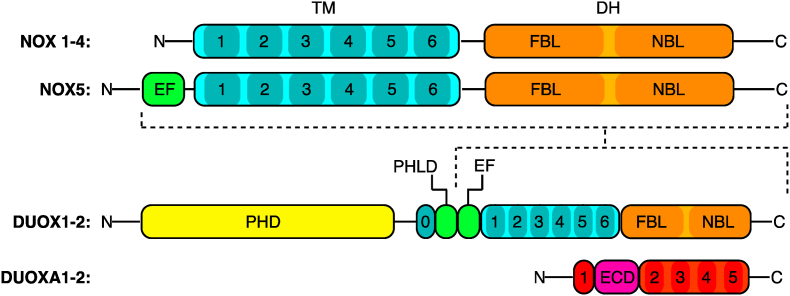
**Primary sequence structure of the NOX enzyme family and DUOXA maturation factors.** The simplest NOX enzymes (NOX1-4) consist of a conserved 6-helix transmembrane (TM) domain that is membrane embedded and an N-terminal dehydrogenase (DH) domain located intracellularly. The DH domain consists of two lobes that bind FAD cofactor (FAD Binding Lobe, FBL) and NADPH substrate (NADPH Binding Lobe, NBL). NOX5 is unique as it contains four N-terminal calcium-binding EF hand domains (EF). The dual oxidases (DUOX1/2) are significantly larger and contain extracellular peroxidase homology domain (PHD) followed by a unique transmembrane helix (0) and, novel to the DUOX enzymes, Pleckstrin homology-like domain (PHLD) as well as two EF-hand domains. DUOX1 and DUOX2 rely on maturation factors DUOXA1 and DUOXA2, respectively, which are a small transmembrane protein that is contained in DUOX1 cryo-EM structures, it consists of an extracellular domain (ECD) and five transmembrane helices. Black outlines define structural domains and darker shading indicates transmembrane helices or structured lobes in the DH domain. Dashed lines indicate the region of DUOX1/2 that is related in domain architecture with NOX1-5.

As membrane-associated proteins, all NOX enzymes consist of a cytosolic dehydrogenase (DH) domain and 6-pass-transmembrane heme-coordinating transmembrane (TM, blue in Fig. 1) domain [1,15]. The DH domain (orange in Fig. 1) comprises an N-terminal lobe that binds the FAD co-factor (FAD Binding Lobe, FBL) and a C-terminal NADPH binding lobe (NBL). The calcium-dependent NOX5 and DUOX1/2 enzymes contain four and two calcium-binding EF-hand (EF) domains, respectively, N-terminal to the TM domain [16]. Both DUOX1/2 contain an N-terminal peroxidase-homology domain (PHD), an additional transmembrane helix (0) and a pleckstrin homology-like domain (PHLD). The activity of the majority of NOX enzymes is initiated by binding of specific proteins, such as p22phox, Rac, and others, whereas NOX4 is the only isoform proposed to be constitutively active as well as negatively regulated by ATP binding [17,18]. Specifically, DUOX1/2 stability and subcellular trafficking is dependent on co-expression and complexation with their corresponding maturation factors DUOXA1/2, [19]; [20] which will be discussed in the context of the DUOX1-DUOXA1 dimer structures (Section 4).

## 3 NADPH substrate binding to the dehydrogenase (DH) domain

**Fig. 2:**
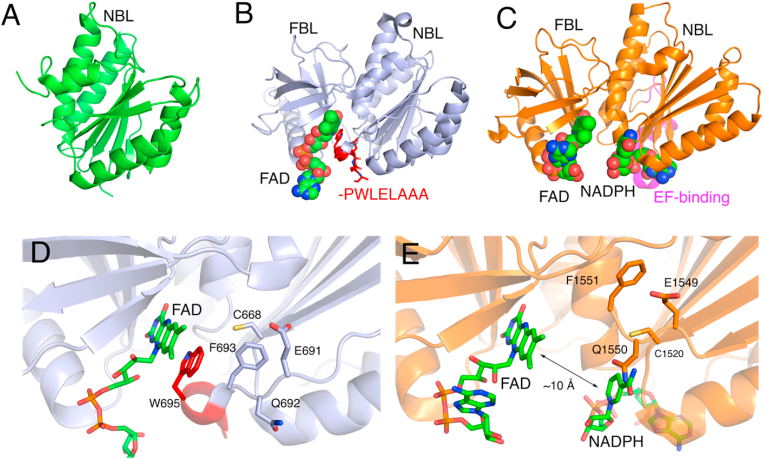
**Substrate binding to the Dehydrogenase Domain (DH) of the NADPH Oxidases.** A) human NOX2 NADPH Binding Lobe (NBL) of the DH Domain (PDB ID 3A1F). B) csNOX5 DH domain containing FAD Binding Lobe (FBL) with bound FAD (green) and C-terminal mutant PWLELAAA (red) (PDB ID 5O0X). C) DH domain from human DUOX1 full-length structure with bound NADPH and FAD (green) (PDB ID 7D3E). D) Zoom in of FAD binding side of B). E) Zoom in of FAD and NADPH binding site of C). (For interpretation of the references to colour in this figure legend, the reader is referred to the Web version of this article.)

**Fig. 3:**
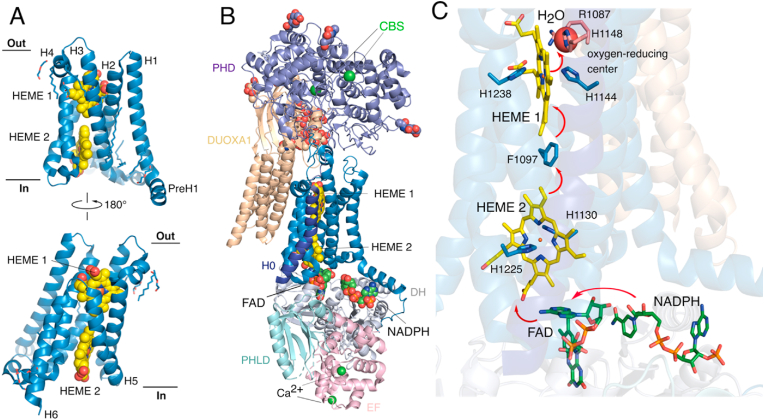
**The transmembrane domain facilitates electron transfer from the dehydrogenase domain to the O**_**2**_**-reducing center.** A) The csNOX5 X-ray crystal structure of the transmembrane (TM) domain showcasing the anchoring of Fe-heme cofactors (yellow spheres) into the plasma membrane in a six-transmembrane ɑ-helix domain (PDB: 5O0T). B) The TM domain in context of the human high calcium (active) full-length DUOX1-DUOXA1 complex structure from Cryo-EM. This structure shows the TM domain as central within DUOX1 and complexed in several domain-domain interactions that enable ET through the plasma membrane. The PHD domain completely covers the extracellular surface of the TM domain and forms protein-protein interactions with the extracellular domain of DUOXA1 (PDB: 7D3F). C) Arrangement of NADPH substrate and flow of electrons (red arrows) from FAD co-factor to Heme 2 followed by electron hopping through F1097 aromatic side chain to Heme 1 and reduction of O_2_ at the oxygen-reducing center as defined by a highly ordered water molecule (the red sphere in panel C). (For interpretation of the references to colour in this figure legend, the reader is referred to the Web version of this article.)

An initial deposited X-ray crystal structure of human NOX2 defined the overall fold of the NADPH binding lobe (NBL) of the DH domain (Fig. 2A). Pioneering efforts by Mattevi and co-workers reported the complete DH domain X-ray crystal structure from *Cylindrospermum stagnale (cs)*NOX5 defining the FBL with bound FAD as well as the NBL containing a C-terminal mutant peptide (red -PWLELAAA in Fig. 2B) [12]. The *cs*NOX5 DH domain NBL fold is practically identical to the NBL of NOX2 (Fig. 2A) as well as the full DH domains characterized in the full-length DUOX1 complexes determined with Cryo-EM (Fig. 2C) [13,14]. Definition of the NADPH binding mode is prevented in the DH domain due to the inclusion of a stabilizing C-terminal insertion -PWLELAA mutation (red in Fig. 2B&D), which positions a non-natural Trp695 in the NADPH binding site adjacent to FAD. More recently, cryo-EM structures of DUOX1-DUOXA1 complexes contain additional structural information on DH domain architecture, including NADPH binding. Structural studies by Sun show bound NADPH (PDB: 6WXV), but lack the nicotinamide group due to insufficient electron density [13]. Cryo-EM structures of human (h)DUOX1-DUOXA1 complexes from Chen and co-workers define the complete NADPH molecule expectedly anchored to the NBL with the C-terminal residues above the nicotinamide as well as previously unresolved sections, such as the EF-hands binding segment (Fig. 2C and E) [14,21]. With respect to function, the NADPH nicotinamide and FAD flavin in this structure are separated by a significant distance that is not conducive for ET (∼10 Å, Fig. 3E) implying that a structural change is necessary for catalysis. Given the high degree of conservation of the C-terminal residues, and their apparent flexibility, this segment may play a role as a regulatory toggle switch by influencing the position of NADPH that allows for efficient ET [12,22]. Furthermore, since NADPH-competitive inhibitors represent promising drug candidates, including VAS2870 that forms a covalent bond with the conserved C668 cysteine (*cs*NOX5 sequence numbers) [23], these collective structures represent efficient starting points for structure-guided drug design.

## 4 Coupling NADPH and O_2_ redox through electron transfer in the transmembrane domain

All NOX enzymes comprise a six-pass transmembrane ɑ-helix domain that complex two b-type hemes responsible for transferring electrons from NADPH through the cell membrane [15,22]. The first structure of the *cs*NOX5 TM domain was solved with X-ray crystallography defining the fold of the transmembrane helices and the Fe heme cofactors [12] (Fig. 3A). Subsequent cryo-EM studies showcased the TM domain as the central focal point in the DUOX1-DUOXA1 complex making interactions with nearly every component of the full-length DUOX1 protein as well as DUOXA1 (Fig. 3B) [13,14]. The TM domain appears virtually identical in all available TM-containing NOX enzyme cryo-EM structures and csNOX5 crystal structure indicating that the TM domain is structurally rigid. The oxygen-reducing center can be identified by a highly ordered water molecule bound by two histidine residues and an arginine adjacent to the Fe-atom consistent with an outer-sphere ET process. Both mouse and human DUOX1 structures contain the PHD at the extracellular surface, however without bound hemes but cation binding sites (CBS) [13,14]. Expectedly, the PHD domain is located at the extracellular surface covering the outer surface of the TM domain and makes contacts with the extracellular domains of DUOXA1. However, several questions remain regarding this domain as no hemes are found in these domains, as consistent with previous recombinant proteins studies [24], and their binding sites are defined by cation binding sites (CBS in Fig. 3B). Functional information from site-directed mutagenesis of residues within the CBS prevents proper hDUOX1-DUOXA1 assembly suggesting a role for PHD in protein stability [14]. Additionally, previous studies have implicated the potential roles of protein-protein intermolecular cysteine disulfides bonds in protein maturation and oligomerization [25], however no such disulfides are observed in the cryo-EM complex structures (Section 5). It seems plausible that the PHD may function to promote hydrogen peroxide production from DUOX1 via superoxide dismutation by delaying diffusion from the TM domain oxygen-reducing center, which has likewise been proposed in the case of the extracellular E-loop of the NOX4 TM domain [26]. Importantly, the hDUOX1 high calcium cryo-EM structure showcases all components in the context of the active state needed to describe the flow of electrons from NADPH to the oxygen-reducing center. Accumulated evidence from the DUOX1-DUOXA1 cryo-EM studies indicate that the DH domain is dynamic, and stabilization of the DH-TM domain interface is critical for activation (Section 5) [13,14]. Therefore, the domain-domain interfacial association of FAD and Heme 2 represents the most important step in the catalytic ET cascade responsible for NOX activity. Additionally, an aromatic amino acid side chain (Phe in DUOX1, Trp in csNOX5) is present in all available TM domains indicating the role of electron hopping to facilitate rapid ET rates across the long intramembrane distance from NADPH to O_2_ [27].

## 5 Structure-based regulation of DUOX1: A prototype for understanding the NOX family enzymes

**Fig. 4:**
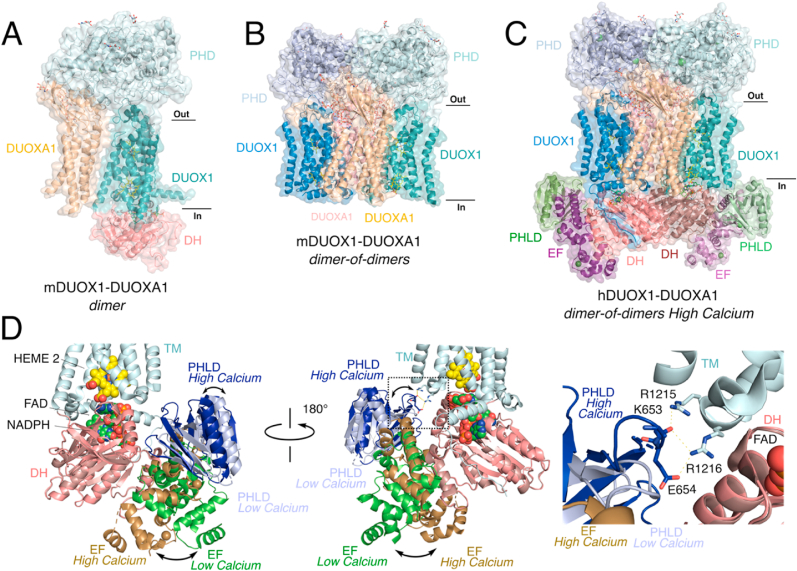
**Factors governing the flexibility of the cytosolic DH domain are key in DUOX1-DUOXA1 activation.** A) 1:1 heterodimer, “dimer,” mouse DUOX1-DUOXA1 cryo-EM structure with structured DH domain (PDB: 6WXV). B) 2:2 dimer of heterodimers, “dimer-of-dimers,” mouse DUOX1-DUOXA1 structure with no structured DH domain indicating enhanced flexibility of the DH domain in this oligomer (PDB: 6WXU). C) “dimer-of-dimer” structure of human DUOX1-DUOXA1 at high calcium with well-ordered DH, two EF-hand domains (EF), and PHLD domain (PDB: 7D3F). D) Overlay of low (PDB: 7D3E) versus high calcium (PDB: 7D3F) DUOX1-DUOXA1 “dimer-of-dimer” structures. The most dramatic structural differences occur in the EF-hand domains and PHLD that reinforce the DH domain in proper domain-domain interface to facilitate effective ET from FAD to Heme 2. Insert highlights high calcium-specific interactions formed as a result of EF-hand and PHLD movement that reinforces the DH domain in the active conformation.

Full-length cryo-EM structures of mouse and human DUOX1-DUOXA1 have allowed for the most comprehensive picture of NOX structure and function, including the association of DUOXA1 maturation factor as well as domains unique to DUOX, including the PHD, two EF-hands (EF), and pleckstrin homology-like domain (PHLD). Purification of mDUOX1-DUOXA1 complexes yields either a 1:1 heterodimer (“dimer” in Fig. 4A) or a 2:2 dimer of heterodimers (“dimer-of-dimers” in Fig. 4B and C). The most significant difference in these structures is the apparent flexibility of the cytosolic domains. In the study by Sun, DH domain flexibility depends on the dimer status of mDUOX1-DUOXA1 complex since it is resolvable in the dimer but not dimer-of-dimer (Fig. 4A versus B) [13]. Generally, the more flexible a domain the weaker electron density and local resolution for that domain, often preventing accurate placement of atoms. On the other hand, both active and inactive hDUOX1-DUOXA1 cryo-EM structures by Chen and co-workers are characterized as dimer-of-dimers and the electron density maps allow for modeling of all intracellular domains (Fig. 4C) [14]. Both studies indicate that the cytosolic DH domains of DUOX1 are dynamically flexible and can be rigidified through dimerization [13] or binding of calcium [14]. A closer examination of the structural differences between the high and low calcium hDUOX1-DUOXA1 complexes reveals the direct impacts of calcium-dependent EF-hand conformation on DH domain stabilization (Fig. 4D). The low calcium structure shows the EF-hands in an extended conformation away from the DH domain (Fig. 4D green), which are brought into contact with the DH domain through a large scale (∼40 Å) swing upon calcium binding (Fig. 4D brown). This movement of the EF hand domains additionally induces a tilt in the PHLD toward the TM domain producing new salt bridge interdomain interactions (light blue versus dark blue in Fig. 4D). The influence of these calcium-dependent structural changes straightforwardly aids to rigidify the DH domain and very likely allow for effective ET from FAD to HEME 2 (Fig. 3C) [28]. Importantly, while there are no structural changes to the DH domain in the hDUOX1-DUOXA1 complex structures, the low local resolution of the DH domain in the low calcium structure is consistent with a more dynamic DH domain and correspondingly higher resolution in the high calcium structure supports the enhanced domain stability in the high calcium structures [14]. It should be noted that the cellular relevance of DUOX1-DUOXA1 dimer-of-dimers is unclear and may be a consequence of producing well-behaved purified and cryo-EM amenable protein complexes. Nevertheless, the collective insights from structural biology indicate that DH domain flexibility is controlled in a structure-based mechanism that enables NOX activity by stabilizing the DH and TM domains.

## 6 Conclusions

Only recently has information on the structural biology of the NOX enzyme family been made available allowing for the visualization of many critical elements that influence their well-recognized biological functions. A combination of X-ray crystallography and cryo-EM have defined catalytic DH and TM domains enabling for atomic-resolution detail of ET from intracellular NADPH to extracellular O_2_. Important insights into NOX enzyme activity are evident from inspections of variations in the apparent flexibility of the DH domains of DUOX1-DUOXA1 complexes and their correlation to altered conformations in accompanying cytosolic domains. This general depiction of DUOX1 activation by structural stabilization of the DH-TM interface can be applied across the NOX enzyme family as well-established protein-protein interactions are known drivers of activity likely by enabling proper rigidification of otherwise flexible cytosolic domains. Despite these broad implications, comprehensive structural information on these enzymes is lacking further motivating structural studies to definitively characterize the molecular mechanism of relevance to each NOX enzyme and their roles in human health and disease.

## Declaration of competing interest

DEH and AvdV are listed on US Patent No. 10,143,718, “Covalent inhibitors of Dual Oxidase 1 (DUOX1)”.
